# Side-Channel Sensing: Exploiting Side-Channels to Extract Information for Medical Diagnostics and Monitoring

**DOI:** 10.1109/JTEHM.2020.3028996

**Published:** 2020-10-06

**Authors:** Aaron Spence, Shaun Bangay

**Affiliations:** School of Information TechnologyDeakin University 2104 GeelongVIC3216Australia

**Keywords:** Cybersecurity, diagnostics, medical, monitoring, POC, point-of-care, sensing, sensors, side-channel, signal processing, smartphones, survey, wearables

## Abstract

Information within systems can be extracted through side-channels; unintended communication channels that leak information. The concept of side-channel sensing is explored, in which sensor data is analysed in non-trivial ways to recover subtle, hidden or unexpected information. Practical examples of side-channel sensing are well known in domains such as cybersecurity (CYB), but are not formally recognised within the domain of medical diagnostics and monitoring (MDM). This article reviews side-channel usage within CYB and MDM, identifying techniques and methodologies applicable to both domains. We establish a systematic structure for the use of side-channel sensing in MDM that is comparable to existing structures in CYB, and promote cross-domain transferability of knowledge, mindsets, and techniques.

## I. Introduction

New avenues for cheaper and ubiquitous medical diagnostics and monitoring (**MDM**) solutions exist due to the continued advances in ‘smart’ devices; smartphones, wearables (e.g., watches, apparel), and increased variety in the sensors they support. Ubiquitous sensing solutions are viable alternatives to traditional MDM devices with the advantage of being cheaper to produce, allow for anytime, any where monitoring for patients, and removing the need to frequently visit specialist medical facilities. While some of these devices are equipped with sensors suitable for MDM applications such as for electrocardiogram (ECG) and photoplethysmography (PPG) readings [1], [2], and serve as diagnostic devices [3], we believe that direct digitisation of a gold standard MDM device does not realise the full potential of these platforms. This literature review presents the emerging trend of using outside-the-box strategies to turn a computational sensing platform such as a smartphone into a versatile toolbox for MDM.

This article explores the concept of *side-channels: the unexpected leakage of information from a physical system via hidden or unknown channels*. There is increasing evidence that ***side-channel sensing*** is being used to create innovative and effective MDM solutions using generic sensing platforms, but with each solution representing a custom and ad-hoc use of side-channels developed in isolation, and without explicit recognition of side-channels. The field of cybersecurity (**CYB**) does utilise side-channels formally and systematically to exploit observable information leakages of electronic target systems to reveal target information contained within, through established processes within its side-channel attacks frameworks [4]–[8]. By analogy [9]: a house with a quality front door lock (e.g., AES encryption) protects against burglars trying every key (a brute force attack) or picking the lock (a cryptographic attack) but is vulnerable to a smashed window (a side-channel). A typical utilisation of side-channels within CYB involves recovering sensitive information (e.g., passwords, media consumption) by analysing changes in signals such as power consumption [10] that are not obviously related to the targeted information. This review compares the approach taken in both CYB and MDM, identifies opportunities for knowledge transfer between these domains, and classifies the techniques employed to benefit from the mindsets employed in each field.

While MDM does not explicitly recognise the concept of side-channels, a plethora of solutions utilise principles employing modality transformations and side-channels within the human body to facilitate novel, non-invasive solutions. These solutions reproduce established gold standard medical diagnostics using low-cost hardware, software-based signal analysis, and the opportunities provided as a result of exploitation of side-channels [11]–[13]. For example, measurement of a common physiological state such as heart rate traditionally achieved via ECG or stethoscope can also be monitored using a variety of side-channels such as sound within the ear canal [14], and PPG via video of the face [15], [16]. An entirely novel approach monitors the oscillations of the chest using WiFi [17].

Mindsets differ between MDM and CYB. CYB operates with mindsets that revolve around the concept of an *adversary*: algorithms that acquire target information through exploitation of the correlation between the target system’s internal operations and acquired leaked signals from a myriad of modalities [4], [18]. Adversaries are countered to protect against access to target information via defence mechanisms (e.g., encryption, leakage countermeasures), or conversely, adversaries are purposely deployed to ‘attack’ the target system by outmanoeuvring said defences. Adversaries require a mindset of cunning innovation, repurposing available tools or exploiting subtle opportunities to support diagnostic processes. MDM instead views the signals leaked by side-channels as opportunities to embrace, as opposed to being ‘flaws’ in the system that compromises security.

While MDM and CYB have very different approaches and objectives, the goals of this article are to:

• Identify a systematic basis for use of side-channel concepts in MDM and compare against approaches in CYB
• Identify techniques and methodologies applicable to both CYB and MDM
• Derive principles of side-channel sensing used in both CYB and MDM that allow transfer of strategies and techniques between the domains, and to identify opportunities for further development DONE - Ideally have the goals clearly listed (looks like new goals are being added as required). Rather start with the goal of comparing side-channel concepts in MDM and CYB, and identifying opportunities for transfer between these domains.

**FIGURE 1.**
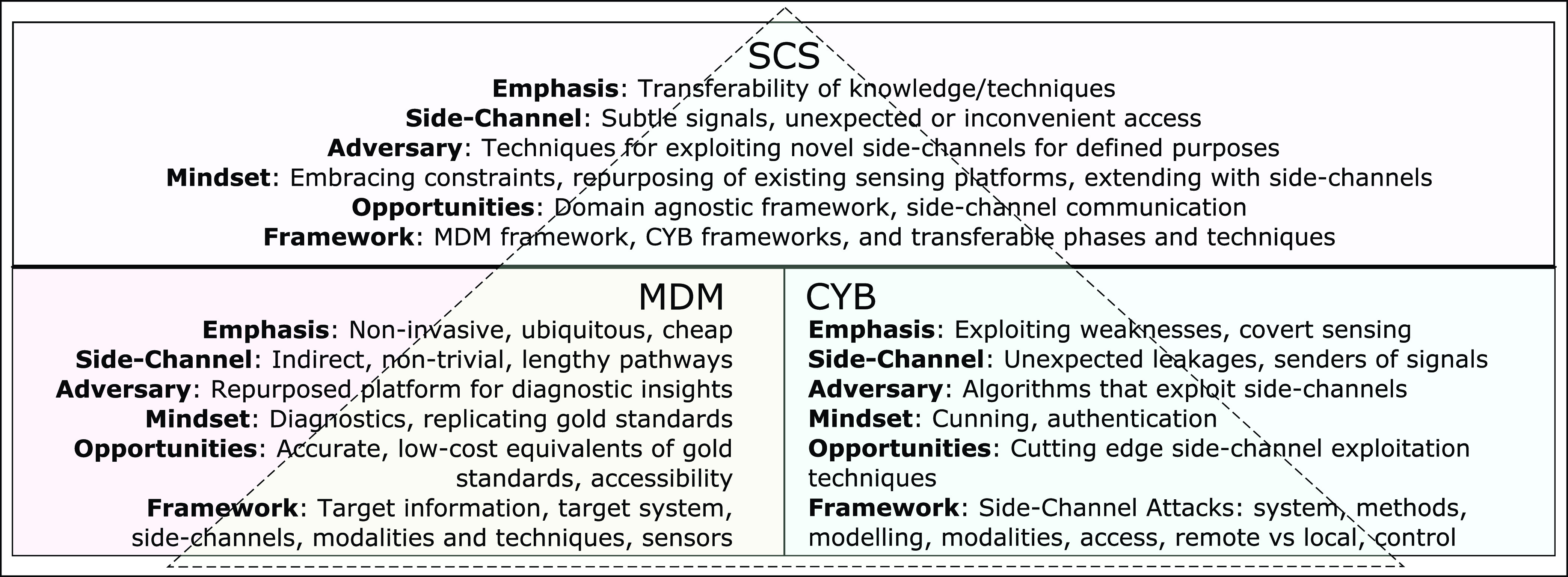
Summary of paper contributions, demonstration of the relationship between side-channel sensing concepts of medical diagnostics and monitoring (MDM) and cybersecurity (CYB), and generalising these concepts to apply to both domains.

The contributions of this article are summarised in Figure 1:

1) First to reveal the widespread use of side-channel sensing within MDM
2) Establishment of an analogous systematic structure for the use of side-channel sensing in MDM that is comparable to existing structures in CYB
3) Establishment of a domain-agnostic side-channel sensing terminology and structure, as depicted in Figure 1
4) Recommendations for cross-domain transferability of knowledge, mindset, and techniques, benefiting both CYB and MDM, and identifying opportunities for future extensions

This article begins with a review of the concept and utilisation of side-channels through the lens of CYB (section II), followed by a similar review of the use of side-channels in MDM (section III). Insights gleaned from these reviews are used to reveal common concepts and techniques that are transferable between them (section IV). This review concludes with presenting a systematic structure for the use of side-channel sensing applicable for use within both domains *(* section V).

## II. Side-Channel Attacks in CYB

This section reviews strategies for side-channel sensing in CYB and the foundational structure and techniques employed within CYB, and introduces potential MDM analogies. CYB enjoys an established side-channel attacks framework for the exploitation of side-channels [4]–[8]. These frameworks are bespoke for the field of CYB and are considered with respect to their relevance to other domains. The key insights and techniques employed by CYB contribute towards this article’s goal of establishing a common structure and transferability between CYB and MDM.

### A. Literature Review Methodology

The CYB specific literature is identified by searching for relevant keywords (side-channels, side-channel attacks, frameworks, cybersecurity, sensors). Additional sources are identified through references in popular press articles that report on unexpected or non-trivial sensing opportunities related to CYB. Sources are selected if they refer to systematic classification of side-channel approaches, or represent recent advances that may not be included in these.

### B. Applications of Side-Channel Attacks in CYB

Utilisation of side-channels in CYB takes advantage of the strong *correlations* between externally measurable signals (e.g., power consumption [5], [10]) and the internal processing of information within the target system is an indicator of information leakage. Any information can be a target, from media consumption [19] on TVs, to eavesdropping and recreating what was printed by a printer [20], [21]. Many CYB originated techniques are applicable to MDM.

A series of measurements of a side-channel constitute an identifiable *signature* which can then be matched against a signature database to identify target information, without having to explicitly decode the side-channel. For instance, since the brightness of a TV image correlates with its power consumption, remote monitoring of the power consumption of a home via smart power meters is a viable side-channel that can identify TV content consumed [19]. Unique power signatures are readily derived from known multimedia content (e.g., movies). The signature in this example consists of time and frequency features derived from training data, and are used to build a random forest based classifier to match power traces against features for known sites. The concept of identifying and matching signatures has applications far beyond the electronic-based target systems within CYB. The human body is a rich source of signals, both internally and externally measured, that contain unique signatures such as breathing patterns [13] that could use similar approaches to characterise lung health.

Entirely remote sensing is possible with modalities that propagate over distances. Screen replication through leaked electromagnetic radiation allows remote viewing of tablet screens [22]. A receiver placed within 2 meters of the target display uses frequency parameters based on prior *profiling* of the target system to decode and display the information received. Such strategies rely on leakage of a signal *beyond its assumed boundary*. Auxiliary channels and out-of-band communication used for authentication tasks are vulnerable to introduced side-channels. Pairing of channels by matching vibrations of neighbouring devices leaks information over acoustic channels [23] that can be extracted using signal processing strategies such as Fourier transforms or *source separation* techniques. Acoustics emanating from printers leak what is being printed [20]. Vibration, acoustics, magnetic fields, and power consumption are modalities emanating along side-channels from 3D printers [21]. Differing modalities can be combined for multivariate solutions where a single sensing point is insufficient, such as the combination of audio and vibration modalities emitted during typing on a keyboard to infer keypresses [24]. Multivariate solutions are particularly effective when paired with *machine learning* due to the wealth of data collected [24], [25]. Despite the various modalities utilised (electromagnetic radiation, vibration, audio), the connection stems from modalities that share the property of propagation over distances. In an MDM context these techniques suggest strategies for health monitoring without the need for physical contact.

Rather than passively sensing signals generated by the target system, an attack can also generate signals, thus *actively sensing*. For example, acoustics can reflect, and travel through a variety of mediums and at varying distances. Taking advantage of these properties, inaudible acoustics emitted from a smartphone can bounce off nearby moving objects, and the corresponding echo can carry target information used to infer the object’s movements [26], [27]. An MDM scenario can be similarly applied to use emitted signals to detect the oscillation of a person’s chest to infer heart and breathing rate [17].

Side-channels can also be used for covert communication and collusion [26], [28]–[30]. These variously employ *modulation* in domains such as those resulting from wavelet or cosine transforms, modulation techniques such as phase modulation and spread spectrum, recovery strategies such as blind detection, and encoding the side-channel to resist attempts to destroy it [31]. New covert channels are commonly introduced for isolated (air-gapped) computers using hijacked and then altered components such as speakers [28] and router LEDs [32] reconfigured to broadcast a modulated signal to a nearby receiver. In these instances, a sensor is *sending* a signal rather than just receiving. A potential MDM analogy might involve encouraging exercise during a physical examination to better identify conditions that cannot be observed in a resting state. It also demonstrates that a sensor can be re-purposed to sense modalities and signals beyond its original design intent, opening up opportunities to sense when under constraint (e.g., where sensor availability is limited).

### C. A Systematic Approach to Side-Channel Attacks in CYB

CYB formalises side-channel usage with its side-channel attacks frameworks [4]–[8], providing definitions, taxonomy, and systematic methodologies and techniques. Born from the seminal works of Kocher *et al.* [10] compromising smart cards using Differential Power Analysis, these frameworks unified the field and demonstrate that side-channel attacks may need only reduce the entropy involved in extracting target information (e.g., secret key) to support attacks.

**FIGURE 2.**
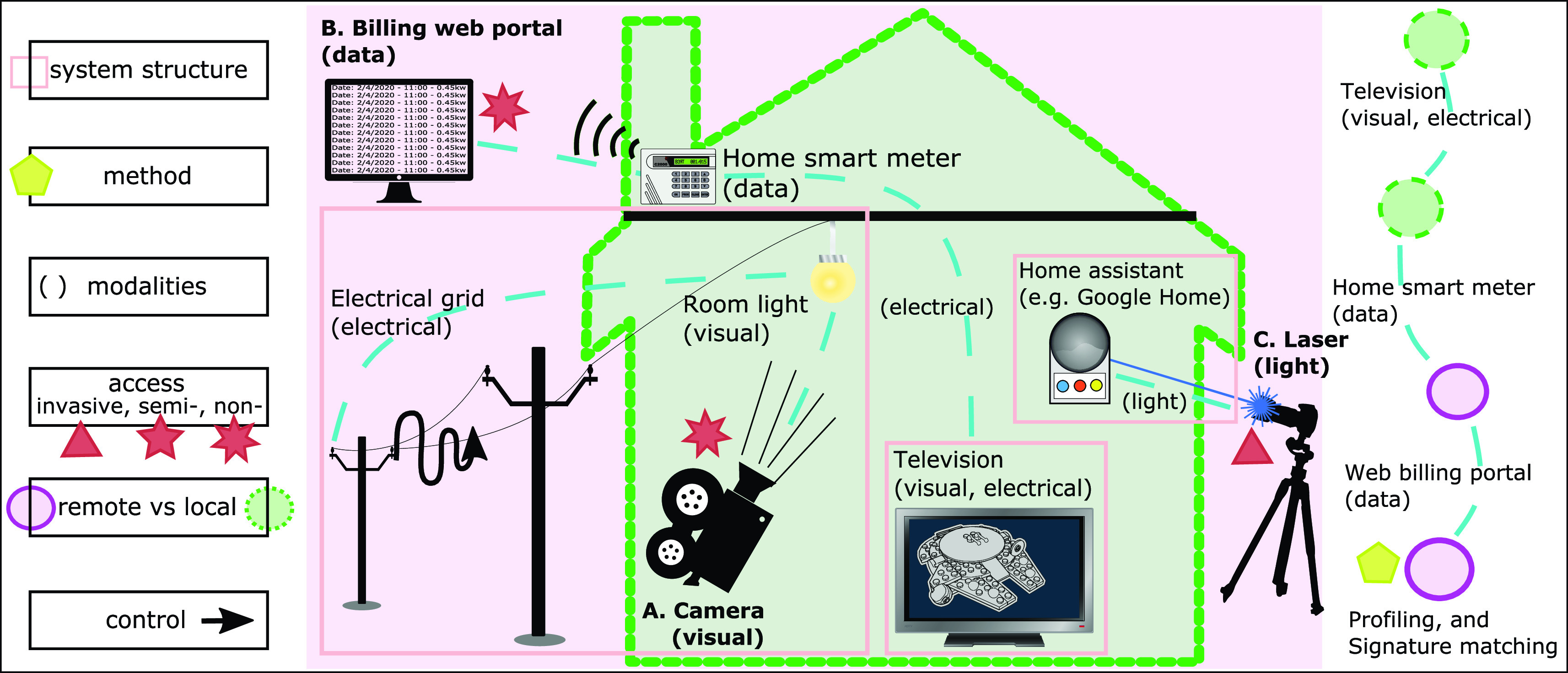
A systematic approach to side-channel attacks in CYB. **Example A:** ‘Geo-location estimation from Electrical Network Frequency signals’ [33]. **Example B:** ‘Multimedia content identification through smart meter power usage profiles’ [19]. **Example C:** ‘Light Commands: Laser-Based Audio Injection on Voice-Controllable Systems’ [34].

The side-channel attack frameworks offer a more structured approach to the use of side-channels with the following concepts common to all instances of side-channel attacks (as depicted within Figure 2):

• **System structure:** the logical components [4]
• **Method:** named attack strategies including *differential analysis correlation*, and *transformation* to frequency or other domains [5], [35]
• **Modelling/Profiling:** uses a training phase to characterise a target system before attacking [4], or for developing a template based on extensive traces [36]
• **Modality:** includes timing [37], [38], power usage [5], [10], electromagnetic radiation [5], [22], magnetic field [30], acoustic [20], [27], visible light [32], infra-red [39], and vibration [21], [23]
• **Access:** level of physical access; invasive, semi-invasive, or non-invasive [5], [40]
• **Remote versus local:** modalities that can be measured from a distance allow for remote sensing [5], [22]
• **Control:** *active* modification to cause side-channel leakage (feeding in a particular input) [5], [8], [34], or *passively* accepting leaked data [5]

Subsequent sections identify examples of side-channel sensing in MDM and develop a corresponding systematic categorisation appropriate to the MDM domain.

## III. Side-Channel Sensing in MDM

Side-channels within MDM denote channels that provide access to target information not directly accessible via available sensors, with an emphasis on non-invasive and outside-the-box solutions. They are indirect and non-obvious, consisting of multiple modality transformations and nodes along the ‘path’ that the information takes from internal to the human body to being received by an external sensor (e.g., camera). Furthermore, side-channels differ from the channels and/or parameters established within gold standard diagnostics. Sensing of side-channels for MDM is used for both monitoring (quantifying a biomarker over a period of time), and for diagnostics (inferring a property of the target information, the cause of the medical condition). Solutions are often built upon existing gold standard solutions since this both supports incremental refinement and helps to validate the solution as acceptable to the medical community. A side-channel sensing version of a gold standard is described by the approach that side-channels are used to acquire access to the target information. Replication of a gold standard exists in four forms: identical channels and parameters [13], identical parameters but different channels [41], different parameters but identical channels [15], [42], or novel solutions with different channels and different parameters [17]. Review of MDM literature that has been deemed to have utilised side-channels, albeit implicitly, is explored in this section.

### A. Literature Review Methodology

The following clarifies the filtering of literature included within this section. Literature searches using keywords such as ‘side-channel’ are not effective because the term is not widely recognised outside of the CYB domain. Creators of MDM side-channel sensing solutions tend to solve their particular problems with reference to previous diagnostic efforts but without reference to comparable sensing strategies. Thus this literature review is seeded starting with popular press articles reporting on unexpected or non-trivial sensing opportunities. The papers and research groups behind these stories are identified and linked through references, citations, and areas of research to identify additional case studies. This strategy is effective in identifying sufficient exemplars to produce a broad, if not exhaustive, survey.

The MDM literature included demonstrates exploitation of side-channels in non-trivial ways (i.e., employing complex signal processing techniques), and target information obtained *indirectly* through pathways and/or modality transformations. We further exclude literature that replicates existing measuring tools or digitisations of clinical scoring systems. We have concentrated on literature that includes as many unique side-channels or target information extraction techniques as possible.

### B. Applications of Side-Channel Sensing in MDM

MDM involves the physician questioning the patient on their *medical symptoms* (patient characteristics that cannot be directly observed by others) and examining for *medical signs* (observable characteristics) [43]. Signs are evidenced through *biomarkers*: “a characteristic that is objectively measured and evaluated as an indicator of normal biological processes, pathogenic processes, or pharmacologic responses to a therapeutic intervention” [44]. Biomarkers are quantifiable and reproducible medical information sources [43], providing leads to either a diagnosis or direction for further investigation. Biomarkers can be directly sensed external characteristics (e.g., skin discolourations), an internal characteristic requiring invasive exploration or extraction (e.g., blood draw) or non-invasive sensing (e.g., bone imaging), or conceptual characteristics (e.g., cognitive behaviours and speech patterns).

MDM long predates electronic devices and thus the quantification of biomarkers was conducted by analogue means and qualitative perception via a physician. Modern medicine introduces devices that quantify information digitally, over extended periods of time, or through sensory modalities not accessible by human senses (e.g., a MRI scan). The fundamental process remains of using a sensor (e.g., physician’s observations, electronic sensors, or diagnostic devices) that quantifies an observable signal (a biomarker) along a channel to infer information about the internal state of the target system (the human body) to make a diagnosis. Modern electronic devices such as smartphones and wearable devices are commonly used for side-channel sensing based MDM solutions [45] due to their ubiquity, affordability, portability, connectivity options (e.g., cellular networks, Bluetooth), and ability to quantify biomarkers across multiple modalities. In MDM, side-channel sensing is less related to finding hidden information and more about sensing under constraint.

Smartphones allow for immediate and continuous monitoring of patients, timely information, and point-of-care diagnostics in a range of environments (e.g., homes, rural communities, developing countries). Smartphones support side-channel sensing based MDM by quantifying biomarkers with their range of embedded sensors; accelerometer [42], [46], magnetometer, gyroscope, light sensor, fingerprint sensor, microphone [13], [47], [48], and camera [11], [16], [49]. Side-channel sensing achieved by re-purposing sensors allows detection of those biomarkers that replicate traditional medical gold standard devices.

Smartphone cameras offer impressive clarity and magnification for quantifying externally visible biomarkers and performing photoplethysmography (PPG). Achievable with a standard camera is the determining of heart rates [16], [41], breathing rates [16], blood pressure [50], atrial fibrillation [12], oxygen saturation [49], classifying the severity of jaundice in newborns via the yellow discolouration of the skin or sclera [11], [51], [52], or even inferring of cognitive loads through visual analysis of biomarkers within pupillary dilation [53].

Third-party tools paired with smartphone cameras are versatile diagnostic instruments [54]. Visual analysis of paper-based immunoassays replace expensive laboratory equipment [55] for detection of osteoarthritis biomarkers [56], quantifying pH levels in sweat and saliva for dehydration monitoring [57], detecting of antibodies in blood plasma using bioluminescence [58], detection of cancerous cells using light diffraction [59], and quantifying salmonella from paper microfluidics [60].

Smartphone microphones can be similarly re-purposed to automatically detect cough frequency [47], [61], [62] using audio collected over a long term, or where fluid in the middle ear can be detected by measuring sounds emitted from the speaker and channelled down a paper funnel [48]. This is typical of sensors being utilised to detect modalities outside of their originally intended capabilities or purpose, for example to diagnose lung health (e.g., cystic fibrosis) through analysis of pressure variations as patients blow into a microphone [13], replicating the gold standard device (a spirometer). A microphone, whose purpose is to convert sound into electrical signals, is now being purposed to quantify pressure variations.

Accelerometers in a smartphone attached to a person detects falls [63], recognise activity [64], measure internal information such as heart rate from the force exerted along the chest cavity when the heart beats [42], and quantify forearm tremors associated with Parkinson’s disease [65].

Wearable devices employ additional sensors for quantifying a wider range of biomarkers; electrodermal activity (EDA), skin and ambient temperature, electroencephalography (EEG), electromyography (EMG), electrocardiography (ECG), and electrooculography (EOG) [1], [2]. Non-invasive wearables attach directly on the skin [66], or are embedded within clothing or accessory devices (glasses, wristbands, watches, and headsets [2], [40]). With a collection of embedded sensors often available within the one wearable, opportunities arise to utilise quantified signals from multiple sensors simultaneously [67], a form of sensor fusion. Wearables focus on monitoring rather than diagnosis as they are unobtrusive and suitable for tracking long-term based medical conditions (e.g., sleep quality, atrial fibrillation).

Having numerous sensors within a single device provides opportunities for multivariate or sensor fusion solutions to improve the accuracy of results, or where results cannot be obtained from single sensor data. For example, established correlations between mental health and multiple factors exist when viewed in combination; daily activity levels (walking, sitting), social engagement (frequency, and whether in-person or virtually), geolocation variations (staying at home versus going outside), and sleep patterns. These factors can all be sensed via a smartphone, using its accelerometer (activity recognition), microphone and app usage (social engagement), GPS and light sensor (geolocation variation), and accelerometer and microphone (sleep patterns) respectively [68].

The utilisation of stand-alone sensors for side-channel sensing for MDM often indicates that the research is in the proof-of-concept and prototyping stages. With a combination of a small microphone and accelerometer placed externally on a patient’s throat, inference of the interior structure and movement of the cartilage and bones can be made, in turn classifying swallowing health [69]. Microwaves reflected off people has been shown to be finesse enough to identify those who may have a gait that exhibits the characteristics associated with shaking palsy (a defining symptom of Parkinson patients) [70]. This type of research provides a rich source of insights into the potentials of side-channels for MDM.

### C. A Systematic Approach to Side-Channel Sensing in MDM

**FIGURE 3.**
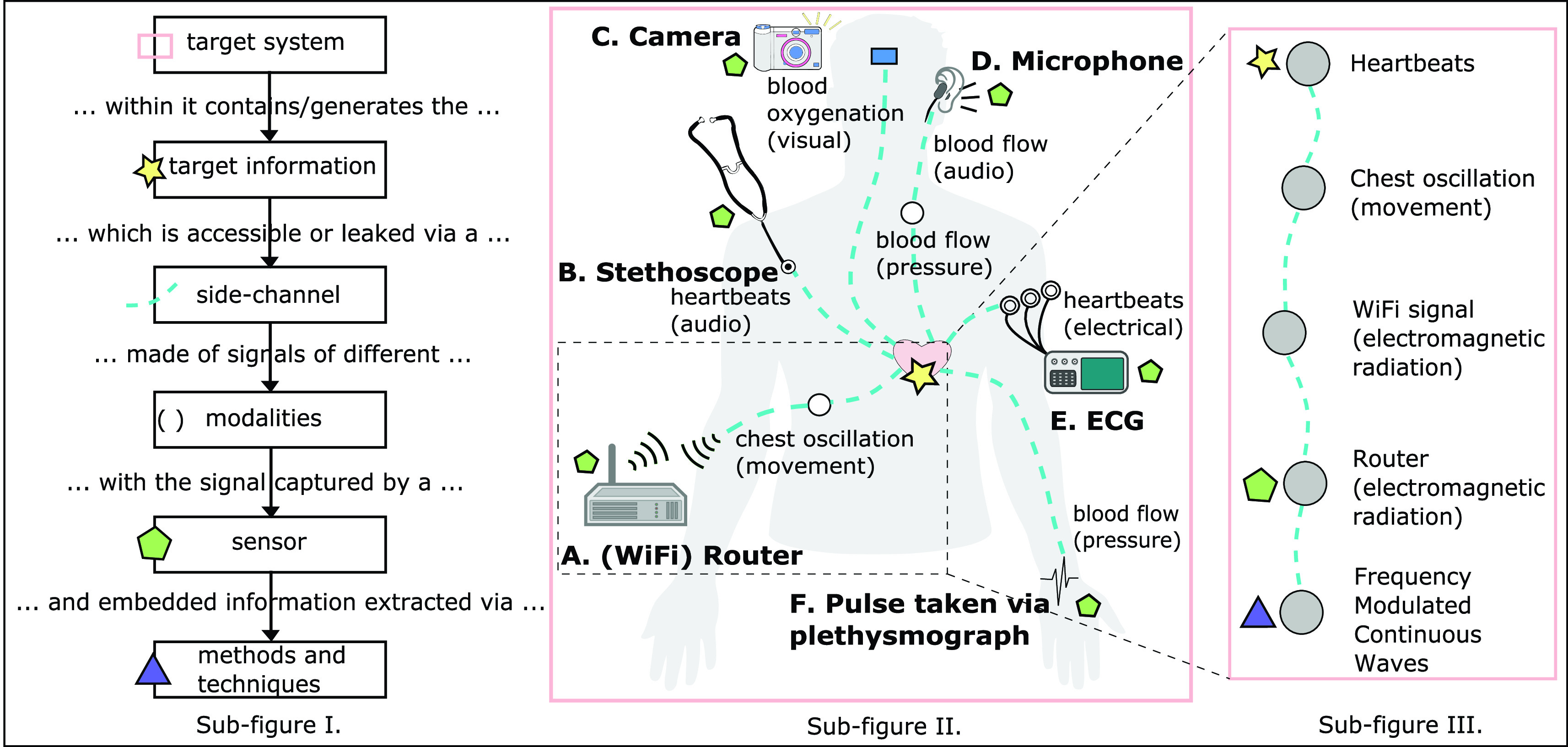
A systematic approach to side-channel sensing in MDM. **Sub-figure I:** Standardised components that make up side-channel sensing, and the flow of information. **Sub-figure II:** Graphical representation of the array of side-channels viable to obtain a single target information (heart rate) within a given target system (human body). **Example A:** ‘Smart Homes that Monitor Breathing and Heart Rate’ [17]. **Example B:** traditional stethoscope. **Example C:** ‘Algorithms for Monitoring Heart Rate and Respiratory Rate From the Video of a Users Face’ [16]. **Example D:** ‘Heartphones: Sensor earphones and mobile application for non-obtrusive health monitoring’ [71]. **Example E:** traditional EEG machine. **Example F:** traditional plethysmograph via pulse. **Sub-figure III: zoom-in** of a single side-channel (Example A) depicting the steps associated with side-channel sensing for a given target system and target information.

Only CYB has any systematic underpinnings with respect to side-channel usage with its side-channel attacks framework (section II-C). Systematic and consistent strategies that can be used to readily build new MDM solutions are absent. In this section the MDM case studies covered are grouped with respect to a more systematic view, and a working terminology, for MDM side-channel sensing (as diagrammed in Figure 3).

#### 1) Target Information

Target information in the context of MDM is the source information that is indirectly sensed through a side-channel, and typically quantifies physical or physiological parameters within the human body (MDM’s target system), for the purpose of a diagnosis or monitoring of a medical condition. Any condition that has associated physiological manifestations (including psychological conditions with physiological biomarkers, such as cognitive load via pupillary response [53]) has the potential to be accessible via side-channels, which can be challenging to measure directly without disturbing or invading the system.

#### 2) Target Systems

The human body is the target system for MDM, an organic system arguably more complex, interconnected, and ‘messy’ than those within CYB. This complexity results in numerous side-channels of varying modalities, making it a rich target for side-channel sensing (as highlighted in Figure 3).

Due to the inherent complexity of the human body as a target system, component categorisation (i.e., breaking down the human body into sub-systems) varies with context. Components identified by location within the body include: back, thorax, abdomen, pelvis and perineum, lower limb, upper limb, and head and neck [72]. Each in turn are comprised of sub-components such as the musculoskeletal components of the back: vertebrae, scapula, vertebral column, and pelvic bone [72]. The spatial coherence of this categorisation supports tracking the physical paths followed by target information through neighbouring components. As an alternative view: the human body consists of several major systems, each of which function through different modalities. These include: nervous (electrochemical), cardiovascular (pressure, chemical), respiratory (chemical, electrical, mechanical force, pressure), urinary (chemical, pressure, visual), gastrointestinal (chemical, pressure), endocrine (chemical, electrical, shape, visual) [73], and integumentary (heat, visual, shape) [74].

Medical conditions may cross between these categorised components, creating opportunities for information leakage. For example, jaundice is a build up of bilirubin in the bloodstream (chemical), possibly due to a compromised liver. The liver, part of the gastrointestinal system, has direct connections to the cardiovascular system. Additionally, jaundice often manifests as a yellow discolouration of the skin, belonging to the integumentary systems (visual) [11], [74]. The foundational principle is that the human body is greatly interconnected and should be viewed as such when considering side-channel sensing for MDM.

#### 3) Side-Channels

A viable side-channel is a pathway between the target information (a biomarker), and a location accessible to a sensor where a quantification can be made (e.g., the skin [15]). MDM includes an additional step whereby a biomarker is evaluated in terms of its reliability in performing a *diagnosis* (further discussed in section V). Established gold standard diagnostic devices rely on proven biomarkers, while side-channel sensing offers the potential to exploit them in new ways or discover new ones.

Biomarkers may exist internally or externally of the target system thus their observability can vary. For example, an external and non-invasive way of sensing red blood cell levels does not presently exist. When internal, direct measurement with ubiquitous, cheap, and available sensors becomes non-trivial with reliance on side or primary channels where the information travels to a more accessible site. Dehydration has an externally available biomarker in the level of pH in sweat [75], quantifiable through collection via a colorimetric strip and analysed through a smartphone camera [57].

#### 4) Modalities

Modalities are highly transformative within the human body, filtering freely from one state to another, such as from chemical to visual in the case of jaundice [11], [52]. Non-invasive measurements are preferred since they do not require healthcare professionals. They observe external biomarkers using sensors such as a camera [11], [41], microphone [13], accelerometer [42], and WiFi [76]. Invasive measurements are achieved by pairing invasive techniques, such as drawing blood, with a separate second stage of sensing such as combining a smartphone’s camera with a modality transformation mechanism such as a colorimetric strip [55], [77].

#### 5) Sensors

MDM solutions employ a spectrum of devices:

• **Stand-alone sensors:** large variety, small size, ubiquitous, and easily embedded into devices [47]
• **Wearables:** real-time, continuous monitoring in direct contact with the body. These can be customised by adding sensors [78] or pairing with attachments [1], [2].
• **Stand-alone smartphones:** ubiquitous, convenient, real-time, continuous monitoring, embedded high-quality sensors, built-in input-process-output capabilities [16]
• **Smartphones with attachments:** expands on smartphone sensing capabilities [45], [59]
• **Wearables/smartphones with remote server:** increased computational capacity for analysis, and opportunities for collating data from multiple sources [1], [78]

Suitability of each option is context specific. For example, the ability to have a wearable to monitor heart rate continuously may be a better option than a smartphone in which the user must take their heart rate manually and periodically. Common between the options, and key to note, is the presence of sensors that can perform quantifications of signals.

#### 6) Phases of Side-Channel Sensing

MDM literature reveals distinct phases for the utilisation of side-channels for the extraction of target information:

1) Discovery: establish that a pathway (i.e, side-channel) from the target information source to a sensing point exists, and that the target information is present on that pathway. This is also where we would quantify the amount of information present on the side-channel.
2) Sensing: with side-channels identified, sensors can acquire the side-channel signals. Obstacles may hinder a sensor’s ability to obtain a signal, such as: target information is internal and thus inaccessible with available sensors [11], [13], signals obtained are too noisy or weak [64], sensors aren’t capable of acquiring the signal [79], or limitations (or preference) on proximity or time-access to the target system exist [16], [41].
3) Extraction: target information is embedded within the sensed side-channel signals. Techniques are employed to extract the target information from the obtained signals.
4) Affirmation: due to the patient-health focused nature of MDM, reliability of results is crucial. The standard method for validation compares output of the MDM solution against a relevant gold standard [11], [13]. Sadly some smartphone apps are poorly validated, with no affiliation to a medical institution, or provide incorrect results [80]. Compliance with relevant regulatory bodies is essential for acquiring independent verification of results, and approval to market the solution [40]. Accuracy of results do not necessarily have to be equal to the gold standard but should be validated to a high degree of accuracy and reproducibility.

Techniques to address each phase, and to overcome obstacles that hinder the completion of a phase, are presented in the following section.

## IV. Analysis and Discussions of Domains

This section compares the approaches used in CYB and MDM with respect to the framework categories for each, including the nature of a side-channel, and the methods and techniques used to access them. Specifically it synthesises the two domains, identifying similarities and opportunities for transferring techniques between domains. The review of the implicit (and hitherto unrecognised) use of side-channels within MDM (section III) has provided insight into their use, with opportunities to extend this work through the identification of key techniques. This section addresses our goal of establishing a common structure between CYB and MDM.

### A. Target Information Within Target Systems

Target information can be digital information (CYB), or a signal associated with some physical process (MDM). Engineered systems (e.g., computers) offer the benefit of knowing in advance that the target information exists within the target system. Access to target information via a side-channel occurs due to leakage, such as the communication of digital information affecting physical properties such as power consumption (CYB), through transformation from one modality to another, or through observation of interactions with the target system. It can also provide value with even a partial recovery of the target information, as this may still provide sufficient insight into a particular execution path (CYB) or allow for a valid diagnosis (MDM). Side-channel sensing in MDM can be approached from a ‘systems’ viewpoint, where the human body is treated as a collection of interconnected components with channels that carry information. This viewpoint is akin to the approach already implemented within CYB, with development of templates/models of the target system to understand the pathway and mechanisms that transport the embedded target information (e.g., encryption key) from a source (e.g., CPU) and the sensing site (e.g., the power consumption) [38].

### B. Side-Channels Within Target Systems

Side-channels exist in target systems from any domain; a key insight expressed and explored in this review. Side-channel sensing retrieves target information that cannot be directly sensed, either because it is actively concealed, inaccessible, or inconvenient to access directly. The different classes of side-channels include those hidden by modality transformations, traversing long sequences of system components, being mixed together with other signals, being hard to measure, needing re-purposing of sensors, or fragmented across multiple channels. The properties of a side-channel share common foundations across both CYB and MDM, however differ in their intent of use and applications. The utilisation of side-channels within CYB is specific to the characteristics of that domain: electronic-based target systems, and a mindset where target systems be ‘attacked’ or outmanoeuvred to overcome its defences, perhaps through invasive sensing or active modifications. CYB approach side-channels as hidden information needing to be recovered through cunning, while MDM exploits the surprising pathways that contain the target information to overcome the natural complexity of human body with its thoroughly connected components. A ‘side-’channel is associated with non-traditional, cheap, and innovative extraction of target information, while the main channel is seen as the existing gold standard approach. Such side-channels have more complex pathways between the target information source and the sensing site during which signals may be mixed, properties manipulated, or modalities transformed. As depicted in Figure 3, side-channels allow access to target information via cheap, ubiquitous, and non-invasive commodity hardware, providing benefits over existing gold standards, albeit through more complex and indirect pathways.

### C. Modalities and Sensing

Side-channel sensing in MDM aims to duplicate, or achieve equivalent results of, gold standard approaches under the constraints of cheap, readily available hardware, without actively modifying the target system. In contrast, CYB solutions are willing to modify the target system, inject signals, run repeated tests, or trigger behaviours that enhance side-channels. The side-channel sensing mindset favours enhancing the functionality of an existing target system through advanced algorithms rather than through replacing physical components.

Side-channel sensing flourishes where information is carried in modalities that facilitate leakage. In MDM, signals propagate over physical media while inside the body, and in electronic systems after sensing. Sensors can be placed inside the target system in some cases for CYB, but breaching the boundary of the body is avoided in MDM. Sensing in CYB is achieved either with custom sensors or, as is common in MDM, using existing sensors on a constrained sensor platform. Modality transformation allows sensors to capture target information that is outside its intended sensing capability. For example, using a microphone to sense pressure variations within the lungs [13], or using speakers to operate as a microphone [28]. Modality transformation is a key strategy in side-channel sensing for both CYB and MDM.

Modalities sensed via multiple sensors can be combined for better information recovery where a single sensing point is insufficient due to low signal levels, where target information is split across multiple channels needing all to recover the target information, or for improving result accuracy [24]. Multivariate solutions are particularly effective when paired with machine learning due to the wealth of data collected [24], [25]. Deep learning can further extend this concept, automating the side-channel discovery and target information extraction process [53].

Information theoretical techniques such as Mutual Information Analysis assists with identifying and quantifying target information present along identified side-channels [81].

### D. Techniques for Extracting Target Information

Existing signal processing strategies focus on primary channels and filter out unwanted content through noise removal. The thesis of this article is that the *noise contains meaningful content in the form of side-channels*. Lack of guidelines on extracting target information from noise results in MDM solutions using ad hoc sequences of features, filters and other individually tuned signal processing stages. Insights from analysis of CYB (section II-B) and MDM (section III-B) accumulate in section V.

### E. Criteria for Side-Channel Sensing

The view of side-channel sensing exploits the following key criteria to applications deemed to have employed side-channels at their foundation:

• Sensing the target information using a modality that is not the target information’s original modality, or sensing a modality that the sensor is not specifically designed for. For example, lung volume is transformed to pressure and then audio levels sensed through a microphone [13].
• The path between the source of target information (e.g., heart rate) and the point at which it is sensed by a sensor contains multiple nodes that may mix in other signals, manipulate properties, or transform the modality of the signal. For example, geographical location is detected based on lighting variations in a video signal which is in turn based on characteristic frequency variation in the local electrical grid [33].
• Sensors for side-channels are software defined, substituting expensive or dedicated sensors with generic devices augmented with non-trivial signal processing techniques. For example, pressure within a closed chamber can be sensed via a generic microphone through inference from recorded audio (e.g., lung capacity and exhaling) [13].

Other properties of the application of side-channel sensing are related to the opportunities that they afford.

• Constrained sensing involves recovering target information while constrained to predefined hardware configurations. A rich source of examples exist in MDM where patients’ own smartphone is used as an inexpensive and readily available solution [61], [82].
• MDM applications of side-channel sensing do not always require the information obtained from side-channels to be identical to the original source information, only that it be sufficient enough to achieve the same diagnostic ability [11], [13].
• The mechanisms of a side-channel need not be entirely understood for successful target information extraction, only that there is an established correlation between the internal mechanisms within a target system and the acquired sensed signals. For example, the mechanism linking sweat pH to dehydration [57] may not be well understood but the established correlation allows one to measure the other.

These insights further highlight that the use of side-channels for the extraction of target information from target systems differs between the domains of CYB and MDM. They also suggest directions for future work within the field of side-channels sensing, particularly towards domains beyond CYB and MDM.

## V. Methods and Techniques for Side-Channel Sensing

MDM solutions are ad-hoc, and are without a framework to build upon, in contrast with CYB and its side-channel attacks frameworks. Both domains however share a common foundation: the utilisation of side-channels to challenge traditional techniques and methodologies to promote more novel, outside-the-box solutions to acquire target information from target systems. Below highlights methods and techniques employed in CYB and MDM literature to acquire signals from side-channels, and to extract the target information embedded within them. Techniques are applicable across both domains regardless of its origin, thus encouraging transferability. Techniques have been tagged with a side-channel sensing phase(s) (introduced in III-C.6) to indicate how and where a technique may be applied within the side-channel sensing process, as well as an indication of the domain that the technique originated from. The tag legend is as follows: Ṁ = MDM, Ċ = CYB, ① = Discovery, ② = Access, ③ = Extraction, ④ = Affirmation.

### A. Modality Transformation

Information is transformed into other modalities. Modality transformations within target systems are driven by internal mechanisms, particularly where the target information is stored or generated, and the pathways from which it may then emanate [50], [69] (Ṁ, ①, ②). Two significant regions of modality transformation are within the target system, and beyond its surface. Lung ailments are assessed with a spirometer (the gold standard device) which measures pressure as a patient exhales into it. Despite the lack of equivalent pressure transducers on a smartphone, interactions between lung, mouth and atmosphere produce audible pressure variations quantifiable with a microphone [13] to estimate the gold standard parameters (volume exhaled) through modelling transfer functions and using machine learning regression techniques (Ṁ, ②, ③). Complex modality transformations allow for more sophisticated and *unexpected side-channel* sensing solutions.

### B. Path Length

The side-channel pathway between the biomarker (i.e., the target information), and underlying cause may be lengthy, involving several nodes. Biomarkers are often not accessible to be quantified *directly* by available sensors. The flow of information from the target information along a side-channel may lead that information to a site on the body accessible by the available sensors (e.g., as skin discolouration which can be quantified with a camera) [11]. The path may invoke modality transformations of the target information between nodes (section V-A) (Ṁ, ①, ②).

### C. Digitising Gold Standards

Many solutions digitise an existing gold standard and consequently inherit structure and constraints from it [69]. Such solutions follow a typical pattern involving quantification of a biomarker using a particular modality and sensor, signal sensing leading to a measurement of the side-channel, deduction of a diagnosis, and verification of results relative to the gold standard (section III-C.6) (Ṁ, ①, ②, ③, ④).

### D. Low Amplitude Signals

Small oscillations of the chest convey heart and breathing rates [17] which are captured in reflected signals emitted from a WiFi transmitter. Such side-channels convey low amplitude signals which are separated through transformation (such as a Fourier transform) and filtering (M, ③).

### E. Context-Based Modelling and Analysis

Extraction of features from a signal is often context-based, where understanding of the target information can dictate what features within the obtained signal are expected to guide model structure and parameter choices. For example, heart beats have a maximum rate of 220 bpm, which can be set as a threshold used in a low-pass filtering stage [14], [16] (Ṁ, ③).

### F. Modelling of the Target System and Its Environment

Exploring channels and modalities in more intricate detail identifies opportunities specific to the context [13], [17]. The gold standard for spirometry (testing lung health) requires placing a mouthpiece in the mouth and exhaling. A smartphone based solution requires the patient to blow into the microphone at arm’s distance [13]. The transfer functions (lung to mouth, mouth to microphone, sound to pressure) are modelled to construct procedures to recover target information (Ṁ, ①, ③). The addition of statistical models such as a Hidden Markov Model can assist with simulating the target system via obtained signal(s) data [62] (Ṁ, Ċ, ①, ③). Modelling/Profile attacks within CYB build models using a training phase to characterise a target system before attacking [4], or developing a template based on extensive traces [36]. Profiling also includes using a copy of the target system to characterise signals acquired from identified side-channels (Ċ, ①, ③).

### G. Feature Extraction

Feature extraction is a primary precursor for machine learning, or signature based matching (as observed in CYB, section II-B). For example, when detecting a heart beat it is expected there to be the recognisable QRS waves (as per the gold standard), thus when applying side-channel sensing to detect heart beats (whether via ECG or other means [12]), these features can be searched for (Ṁ, ③). Deep learning algorithms (e.g., convolutional neural networks) offer unique potential; the ability to automatically complete the feature extraction stage on given sensed data from side-channels paves way for identifying new side-channels through experimentation [49] (Ṁ, ③).

### H. Machine Learning

As an alternative to context specific signal processing pathways, machine learning following a feature extraction step can be applied where there is a dataset available and known patterns need to be extracted [61] or to perform classification [53], [67], [68], [70] (Ṁ, ③). Machine learning can be a powerful tool in the toolbox of target information extraction techniques, but a thorough analysis of the myriad of machine learning techniques is beyond the scope of this article. Deep learning techniques offer automated target information extraction (Ċ, ③), and even identification of novel side-channels through previously unknown correlations with target information (Ċ, ①), given a sufficient and sizeable training collection of sensed signals [24], [25], [49].

### I. Information Theoretical Approaches

Signals obtained from a target system are almost always a mixture of signals. Information theory approaches such as the field of blind source separation have the objective of separating individual signals from a mixture of signals to extract target information, where all the other information sources can be temporarily regarded as noise [83]. Stateful systems reduce entropy of user input facilitating recovery of side-channels [84]. Mutual information provides a correlation measure to validate presence of a particular side-channel in a signal [21], [81], and allows observation of the signal to reduce entropy or increase the perceived information [85] of the target information (Ṁ, Ċ, ①, ③). Techniques such as Independent Component Analysis, Principal Component Analysis, and Non-Negative Matrix Factorisation have been shown as valid approaches within MDM, CYB and beyond.

### J. Transformations

Individual techniques include signal separation strategies that transform into a space (e.g., via FFT or PCA) that facilitates filtering [17], [41], [61] or feature extraction, such as Power Spectral Density [82] or context specific features such as pulse amplitude, rate and variability [50]. Filter parameter values are set during a calibration phase [11], [12] (Ṁ, ③).

### K. Signal Interference

Signal interference deals with situations where sensors are ineffective and involves purposefully altering the target system through addition, subtraction, or manipulation [55], [79] (Ṁ, ①, ③). An unaltered smartphone camera is not sufficient to quantify pathogens in a blood sample due to the size of the cells. White submicrobeads added to the blood sample tend to congregate with the blood pathogens. Further adding light from the device’s flash supports spectroscopic measurement of the light reflectance off the white submicrobeads and reveals the target information [79] (Ṁ, ①, ③).

### L. Optimal Sensor Placement

Placing sensors at optimal positions is significant for MDM due to the interconnected and noisy nature of the human body (section III-C.2). Sensor placement exploits the existence of physical channels between the sensor location and a source of the target information [64] (Ṁ, ②).

### M. Device Spectrum, and External Tools

A common obstacle to MDM is that the target information is internal to the body and choice of sensors is constrained (e.g., when limited to a smartphone). Extending a device with hardware elsewhere along the device spectrum (section III-C.5) provides access to samples acquired invasively, adapts existing sensors, or adds new ones [55], [79] (Ṁ, ②).

### N. Adopting Mindsets From Other Domains

Adapting mindsets from other domains allows for viewing of target systems from an angle different to what is traditional for that domain, potentially revealing new side-channels or information extraction techniques. The foundation that links CYB and MDM is the compatibility and transferability of their respective mindsets, with both domains attempting to build solutions that extract target information from a given target system through the *novel sensing of side-channels*. The CYB ‘attack’ mindset may prove useful for MDM; encouraging a side-channel sensing based approach emphasising cunning [85]. Covert sensing is not a requirement but unobtrusive sensing methods are still an advantage in many scenarios, particularly within MDM where non-invasive sensing is preferred. Conversely, MDM’s emphasis on sensing using ubiquitous platforms demonstrates that sensing can still be achieved when under constraints (e.g., limited sensor availability), encouraging exploration of lengthy pathways, modality transformations, and outside-the-box thinking.

## VI. Conclusion

This article demonstrates the use of side-channels applied across the domains of cybersecurity (CYB) and medical diagnostics and monitoring (MDM), involving the use of available *sensor* data in a *non-trivial* way to acquire previously unknown, *hidden* or unused *target information* from *target systems*. The range of literature analysed extends on previous interpretations of side-channels to include sensing of subtle signals by exploiting information leakages, signals mixing or routing along diverse paths, and modality transformations exploited by re-purposed sensors (section IV-E).

Traditionally utilised within CYB, this review is the first to formally recognise their use, and potential further use, within MDM that until now were ad-hoc and lacked structure. Both domains have distinctive approaches to side-channel sensing but submit to classification under categories such as target systems, side-channels, modalities, and techniques for acquiring their target information (section IV). Advanced sensing and signal processing techniques underlie most of the examples. Side-channel sensing identified the foundations shared between CYB and MDM, promoting cross-domain transferability of knowledge, mindsets, and techniques (section V). The devious mindset associated with CYB solutions could lead to valuable medical outcomes through outwitting the undocumented and complex biological processes in a human body. Conversely, MDM solutions offer value from their ability to thrive despite sensor availability constraints, and inability to invasively probe or modify the target system, instead relying on lengthy pathway, modality transformations and novel approaches. This differs from existing medical practice that favours incremental advances on traditional solutions. Side-channel sensing solutions tend to favour advanced software solutions in situ over target system modification, which is well suited to finding innovative ways of using existing sensing platforms for medical diagnostics.

Exciting possibilities exist by projecting side-channel sensing opportunities forward, specifically within the domain of MDM (but certainly even beyond). The classification criteria also serve to identify opportunities for further extensions to the field. Some ideas identified include using very large arrays to construct virtual sensors, actuation as well as sensing using side-channels, and manipulation of the target system to introduce or enhance side-channels.
